# Association of Ocular Inflammation and Rubella Virus Persistence

**DOI:** 10.1001/jamaophthalmol.2018.6185

**Published:** 2018-12-27

**Authors:** John A. Gonzales, Armin Hinterwirth, Jessica Shantha, Kaidi Wang, Lina Zhong, Susie L. Cummings, Ying Qian, Michael R. Wilson, Nisha R. Acharya, Thuy Doan

**Affiliations:** 1 Francis I. Proctor Foundation, University of California, San Francisco; 2 Department of Ophthalmology, University of California, San Francisco; 3 Department of Ophthalmology, Emory University, Atlanta, Georgia; 4 Department of Ophthalmology, Kaiser Permanente, Oakland, California; 5 Weill Institute for Neurosciences, University of California, San Francisco; 6 Department of Neurology, University of California, San Francisco

## Abstract

**Importance:**

Metagenomic deep sequencing (MDS) demonstrates that persistent and active rubella virus (RV) infection is associated with Fuchs heterochromic iridocyclitis (FHI).

**Objective:**

To assess the utility of MDS in identifying RV infection in patients with uveitis.

**Design, Setting, and Participants:**

This case series assessed 6 patients diagnosed by MDS with RV-associated uveitis at a tertiary uveitis referral center in the United States.

**Exposures:**

Prior RV infection.

**Main Outcomes and Measures:**

Clinical examination findings, slitlamp photography, corneal confocal imaging, and infectious pathogen genome obtained from RNA sequencing.

**Results:**

Six white men (age range, 36-61 years) were diagnosed with RV-associated uveitis by MDS. Three patients exhibited iris heterochromia associated with their uveitis in classic FHI fashion. The other 3 patients had less classic FHI features and exhibited anterior vitritis. Three patients had in vivo corneal confocal microscopy, with 2 demonstrating stellate keratic precipitates in addition to endothelial infiltration, spotlike holes, and enlarged intercellular boundaries. Of these 3 patients, 1 patient exhibited polymorphism and polymegathism of the endothelial cells.

**Conclusions and Relevance:**

These findings suggest that persistent RV infection is associated with recurrent or chronic anterior or anterior-intermediate uveitis as well as corneal endothelial cell damage. Ophthalmologists should consider RV infection as a potential cause of hypertensive anterior and intermediate uveitis.

## Key Points

**Question:**

What are the ocular findings of patients with the rubella viral genome detected with metagenomic deep sequencing?

**Findings:**

In this case series study, 6 patients with detectable rubella virus RNA in the intraocular compartment exhibited typical and atypical characteristics of Fuchs heterochromic iridocyclitis. Confocal imaging of the cornea revealed endothelial cell alterations in the affected eyes.

**Meanings:**

These findings suggest that patients with persistent intraocular rubella virus infection can present with heterogeneous clinical findings, including endothelial cell damage.

## Teaser

This case series assesses the utility of metagenomic deep sequencing in identifying rubella virus infection in patients with uveitis.

## Introduction

The eye is an immunoprivileged site that can harbor viruses for years.^1^ Chronic infection of the positive-sense single-stranded rubella virus (RV) is a cause of the Fuchs heterochromic iridocyclitis (FHI) phenotype.^2,3,4,5^ Prior reports^3,6,7^ have noted that intraocular fluid obtained from many cases of FHI demonstrate the presence of antibodies to RV, yet reverse transcription–polymerase chain reaction (RT-PCR) has frequently failed to demonstrate the presence of RV RNA. This discrepancy has been suggested to reflect a limited period during which the virus may be detected or persist in the eye.^3,6^

Unbiased metagenomic deep sequencing (MDS) is a high-throughput sequencing approach that can identify all genomes present in a clinical sample. Prevous studies^8,9^ have demonstrated that MDS of intraocular fluid can detect fungi, parasites, and DNA and RNA viruses in patients with intraocular inflammation.^8,9^ We present a case series of patients with rubella-associated uveitis diagnosed with MDS and assess the utility of MDS in identifying these infections.

## Methods

This case series included 6 patients referred to the Francis I. Proctor Foundation, University of California, San Francisco (UCSF) for evaluation of recurrent or chronic hypertensive nongranulomatous anterior uveitis or hypertensive intermediate uveitis with concern for vitreal lymphoma (eTable in the Supplement). Ethical clearance was obtained from the institutional review board at UCSF, and the study adhered to the tenets of the Declaration of Helsinki.^10^ Written informed consent was obtained from all patients, and all data were deidentified.

Five patients (83%) were immigrants to the United States, whereas 1 (17%) was born in the United States before the institution of routine RV vaccination. Two patients exhibited anterior uveitis, whereas the remaining 4 exhibited anterior-intermediate inflammation. Two patients had no prior topical corticosteroid exposure. Five patients had a history of ocular hypertension, with 4 patients having gonioscopy performed that revealed open angles on gonioscopy, although 1 patient featured bridging vessels. Four eyes in 4 patients had corneal sensation examined, with 3 eyes exhibiting reduced corneal sensation. All involved eyes exhibited nongranulomatous keratic precipitates, with 4 having diffuse stellate keratic precipitates. Four eyes from 3 patients exhibited both iris atrophy and iris transillumination defects, 2 patients exhibited iris heterochromia, and 1 patient had no iris defects compared with the contralateral unaffected eye. All patients maintained visual acuity, ranging from 20/20 to 20/60. Two patients had fluorescein angiography performed without vascular leakage or staining of the disc (eFigure in the Supplement).

**Figure 1.**
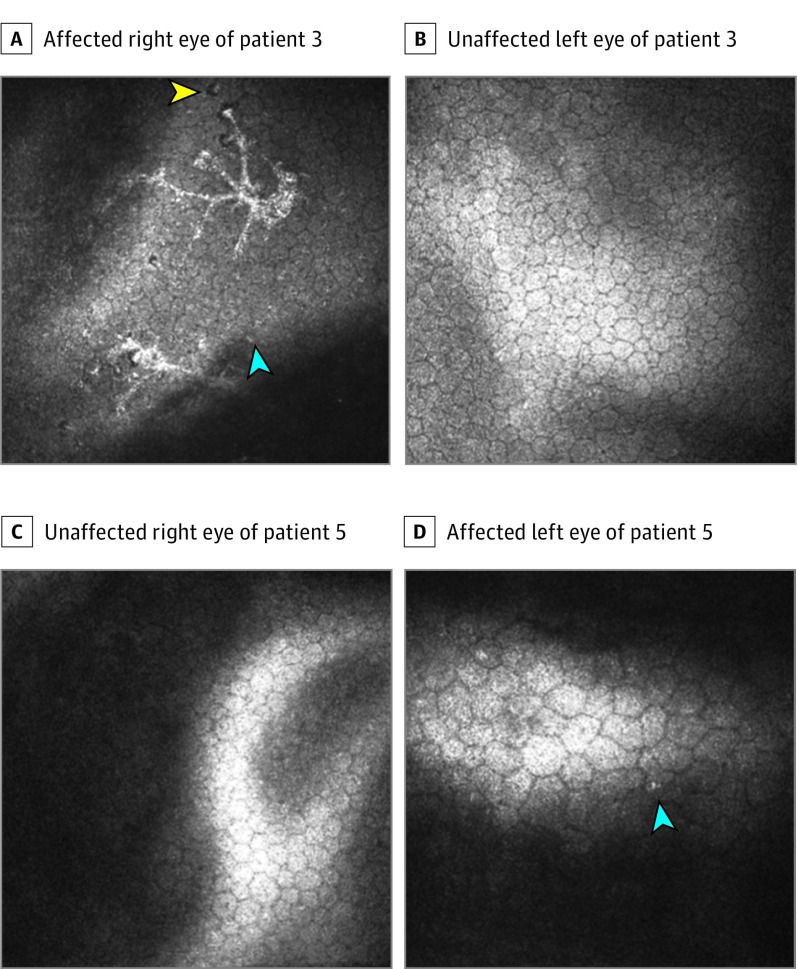
Confocal Images From the Patient Cohort Confocal images of the affected right eye (A) and unaffected left eye (B) of patient 3 showing infiltration of endothelial cells with endothelial infiltration (blue arrowhead) and spotlike holes (yellow arrowhead). Confocal images of unaffected right eye (C) and affected left eye (D) of patient 5 showing polymegathism and polymorphism as well as infiltration of endothelial cells (blue arrowhead).

Three patients received confocal microscopic imaging in our clinic. We found that the affected eyes exhibited spotlike holes, increased intercellular spaces, and infiltration of endothelial cells (Figure 1). In addition, all affected eyes exhibited features of polymorphism and polymegathism compared with the contralateral eye (Figure 1D). Two patients also exhibited stellate keratic precipitates on confocal microscopy, and 1 patient exhibited spotlike holes and endothelial infiltration in the unaffected eye. Similar findings have previously been described for herpes simplex virus–associated endothelial involvement.^11^

All 6 patients had anterior chamber paracenteses performed at the Proctor Foundation, where fluid was submitted for targeted herpes simplex virus 1 and 2, varicella-zoster virus, and cytomegalovirus PCRs, with residual fluid subjected to MDS.^8^

## Results

**Figure 2.**
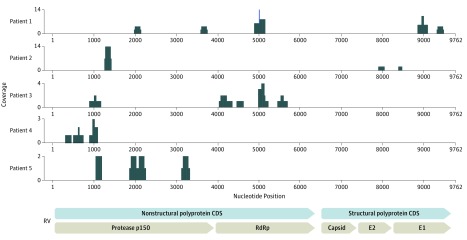
Rubella Virus (RV) Genomes From the Patient Cohort Rubella virus sequences from 5 patients assembled against the reference RV genome. CDS indicates coding sequence; RdRp, RNA-dependent RNA polymerase.

All samples from the 6 white male patients (age range, 36-61 years) tested negative for herpes simplex virus 1 and 2, varicella-zoster virus, and cytomegalovirus by PCR, but all tested positive for RV RNA by MDS. Figure 2 shows the heterogeneous capture of the RV genome regions among samples, reflecting the unbiased nature of the assay. The MDS coverage of the RV genome for patient 6 is described elsewhere.^8^ Genes in the nonstructural coding sequence were detected for all 6 samples, whereas only 3 samples tested positive for genes in the structural coding sequence. Orthogonal testing using RT-PCR for the RV *E1* gene was performed on 2 samples. Only the sample that yielded the highest number of reads on MDS tested positive on RT-PCR with a low cycling threshold of 38, demonstrating the potential utility of MDS in suspected cases of FHI.^8^

## Discussion

The World Health Organization declared RV elimination in the Americas in 2015 as the result of effective vaccination policies.^12^ Because humans are the only host for RV, the prevalence of FHI in the United States was reduced substantially after the introduction of RV vaccination.^13^ Rubella infection, however, remains a threat throughout other parts of the world. Five of 6 patients in this study emigrated from regions where the vaccination policies were not strictly enforced. From a clinical standpoint, asking patients who live outside the United States about their immunization status or checking RV IgG levels is of little help in the workup for FHI because all immigrants are required to have the mumps-measles-rubella vaccine before entry. Furthermore, few patients will remember if they were exposed because only 2 of the patients in our study recalled having German measles as children. Although the theoretical risk of transmission exists because the patient’s RV strain in the eye can be genetically different than that of the vaccine strain,^8^ in the absence of intraocular surgery or globe trauma, the functional risk is likely minimal. In the 3 patients who underwent RT-PCR testing for RV RNA in the tears, nasopharynx, and urine, all samples tested negative for viral genome, indicating that the virus was exclusively localized to the eye.

Molecular diagnostics for intraocular rubella infections are not routinely available in the United States. The Goldmann-Witmer coefficient assay (requiring both intraocular fluid and a serum sample) and the RT-PCR for RV, which are available in Europe, can be limited in scope.^3,7,14^ The RV-directed RT-PCR usually targets a small region (approximately 185-739 base pairs [bp]) of the *E1* gene, which is a small fraction of the 9762 bp of the entire RV genome.^8,15^ However, MDS is an unbiased approach that has the potential to detect any genome region of a particular pathogen in a clinical sample (Figure 2). Furthermore, the unbiased nature of the assay allows for the detection of both common and rare pathogens in minute amounts of intraocular fluid (approximately 20-50 μL) without requiring a priori knowledge, as is the case with pathogen-directed PCR.^8^ In contrast to prior work that used RT-PCR,^3^ this study found that regardless of age, the RV genome can be detected in patients with FHI using MDS. Our findings also demonstrated that anterior chamber paracentesis for RV testing by MDS may be sufficient even if the inflammation is localized mainly in the vitreous cavity of phakic patients.

A major limitation of this study is that the population studied is from a referred group of patients with previously undifferentiated anterior and intermediate uveitis. In such cases, other causes, including syphilis and tuberculosis, must be ruled out. All of the patients had negative treponemal antibody and interferon gamma release assay test results and normal findings on chest radiography.

## Conclusions

In summary, these findings from MDS suggest that inflammation in patients with FHI (in the classic FHI phenotype or anterior-intermediate uveitis) is stimulated by the persistent presence of RV in the eye and that these affected eyes exhibit evidence of corneal endothelial cell damage previously not appreciated without confocal imaging. As other studies^14,16^ have demonstrated, patients with RV uveitis can maintain usable visual acuity.

## Notes

Supplement. **eFigure.** Posterior segment features in rubella virus associated uveitis **eTable.** Ocular features in patients with rubella-associated uveitis
