# Supplementary material for: Association of Ocular Inflammation and Rubella Virus Persistence
Source: JAMA Ophthalmol. 2018 Dec 27;137(4):435–8. doi: 10.1001/jamaophthalmol.2018.6185 (PMC6439711; doi:10.1001/jamaophthalmol.2018.6185)
Supplement: Supplement. — eFigure. Posterior segment features in rubella virus associated uveitis eTable. Ocular features in patients with rubella-associated uveitis [file jamaophthalmol-137-435-s001.pdf]

Supplementary Online Content

Gonzales JA, Hinterwirth A, Shantha J, et al. Association of ocular inflammation and rubella virus persistence. *JAMA Ophthalmol.* Published online December 27, 2018. doi:10.1001/jamaophthalmol.2018.6185

eFigure. Posterior segment features in rubella virus associated uveitis

eTable. Ocular features in patients with rubella-associated uveitis

This supplementary material has been provided by the authors to give readers additional information about their work.

© 2018 Gonzales JA et al. *JAMA Ophthalmology.*

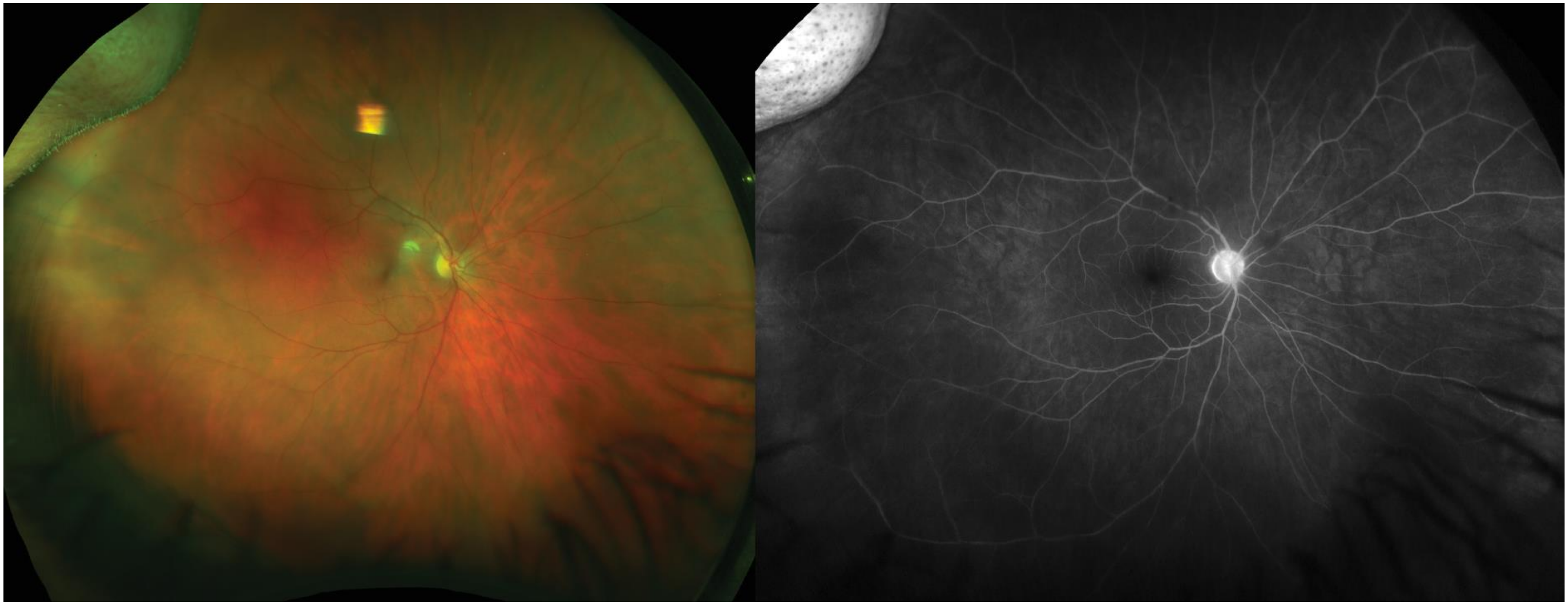

eFigure. Posterior segment features in rubella virus associated uveitis. No patients exhibited features of posterior segment inflammation clinically. Two patients had fundus photography and fluorescein angiography performed which did not exhibit vascular leakage or staining of the optic disc (the right eye of a 36-year-old man with bilateral RV associated anterior/intermediate uveitis shown here). Ultrawide-field fundus photo (left) and late frame ultrawide-field fluorescein angiography (right).

© 2018 Gonzales JA et al. *JAMA Ophthalmology.*

eTable. Ocular features in patients with rubella-associated uveitis.

Patient	Age at AC paracentesis	Sex	Ethnicity	Uveitis Anatomical Classification	Ocular HTN	Gonioscopy	Corneal sensation[1]	Keratic precipitates (KPs)	Confocal Findings	Iris
1	60 years	M	Caucasian (born in US)	A/I	Yes	Bridging vessels	Not available	Small/NG with associated MCE	Not performed	Peripheral iris atrophy; TIDs; heterochromia
2	57 years	M	German	A/I	Yes	Open to SS	Reduced	Stellate; Round/NG	Not performed	WNL
3	61 years	M	German	A	Yes	Open to CB	Reduced	Stellate	Stellate KPs; spot-like holes, enlarged intercellular boundaries; endothelial infiltration	Atrophy with small TIDs
4	56 years	M	Russian	A/I	No	Open to CB	WNL	Stellate inferiorly	Stellate KPs; spot-like holes, enlarged intercellular boundaries; endothelial infiltration	Heterochromia
5	46 years	M	Lebanese	A	Yes	Open to CB	Reduced	NG	Polymegathism, polymorphism	TIDs
6	36 years	M	German	A/I	Yes OU	Not available	Not available	Stellate OU; Large/NG OU	Not performed	Peripheral atrophy OU; iridodonesis OS; TIDs OU

[1]Refers to corneal sensation in affected eye compared to non-affected eye; HTN = hypertension; CB = ciliary body; A/I = anterior/intermediate uveitis; A = anterior uveitis; TIDs = transillumination defects; WNL = within normal limits; OU = oculus uterque (both eyes); NG = non-granulomatous; OS = oculus sinister (left eye); SS = scleral spur; MCE = microcystic edema.

© 2018 Gonzales JA et al. *JAMA Ophthalmology.*
